# Virulence properties of cariogenic bacteria

**DOI:** 10.1186/1472-6831-6-S1-S11

**Published:** 2006-06-15

**Authors:** Howard K Kuramitsu, Bing-Yan Wang

**Affiliations:** 1 Department of Oral Biology, State University of New York, Buffalo, NY 14214, USA

## Abstract

The importance of *Streptococcus mutans *in the etiology of dental caries has been well documented. However, there is growing recognition that the cariogenic potential of dental plaque may be determined by the composite interactions of the total plaque bacteria rather than solely the virulence properties of a single organism. This study will examine how the interactions of *S. mutans *with other biofilm constituents may influence the cariogenicity of plaque samples.

In order to begin to investigate the effects of nonmutans streptococci on the cariogenic potential of *S. mutans*, we have examined the effects of *Streptococcus gordonii *on the virulence properties of the former organisms. These studies have indicated that *S*.*gordonii *can attenuate several potential virulence properties of *S. mutans *including bacteriocin production, genetic transformation, and biofilm formation. Therefore, modulation of the interactions between plaque bacteria might be a novel approach for attenuating dental caries initiation.

## Introduction

Although the incidence of dental caries has declined over the past few decades in highly industrialized countries, this disease is still a significant public health problem [1]. Despite the recognition of the importance of mutans streptococci, principally *Streptococcus mutans*, as primary etiological agents of caries and the role of environmental factors such as diet on this disease [2], no novel anticaries therapies based upon this information have been recently incorporated into routine dental practice. Intensive investigation into the virulence of *S. mutans *has identified a number of properties of these organisms which are likely to be important in cariogenesis including: sucrose-dependent biofilm formation, relatively high aciduricity, and potent acidogenesis [2]. However, it is now recognized that other plaque constituents may also be relevant to caries initiation including nonmutans streptococci [3] and alkaline bacteria [4], as well as recently identified novel organisms [5].

As elegantly emphasized by Kleinberg [4], it is apparent that the interactions between cariogenic bacteria such as *S. mutans *with other nonmutans plaque constituents can modulate the cariogenic potential of a specific plaque sample. A more careful examination of this hypothesis would require a "systems biology" approach to dental plaque, *i.e*., determining how the individual plaque constituents interact to produce the resulting properties of a biofilm community. Therefore, in order to investigate how such interactions could affect the cariogenicity of a plaque sample, we have begun to investigate the effects of nonmutans streptococci on the cariogenic properties of *S. mutans*. Earlier studies have suggested an inverse relationship between the presence of *S. mutans *and strains classified as *Streptococcus sanguis *(including *Streptococcus gordonii*) in human dental plaque [6]. Therefore, an understanding of the molecular basis for such interactions could prove useful in designing new strategies to control human dental caries.

## Methods

### Bacterial strains

*S. mutans *strains GS5, LT11, NG8, and BM71 as well as *S. gordonii *Challis were routinely cultured in Todd-Hewitt broth (THB; Difco, Detroit, MI) and maintained on Tryticase soy agar plates. The construction of the Challis mutant BYW1 was recently described [7].

### Transformation

Genetic transformation of *S. mutans *strains with *Escherichia coli*-streptococcal shuttle plasmid pPGS479 (Wang and Kuramitsu, unpub. results) in broth was carried out essentially as routinely carried out in this laboratory [8]. When mixed cultures of *S. mutans *and other oral streptococci were examined for transformation, the transformed cells were plated onto Mitis salivarius agar plates containing erythromycin (Erm, 10 μg/ml) to differentiate between *S. mutans *and nonmutans streptococci.

For transformation in biofilms, mixed biofilms were initially formed with *S. mutans *strains and *S. gordonii *Challis (10^5 ^cfu) in 0.10 ml one-fourth diluted THB-0.1% glucose containing 0.01% bovine mucin (Sigma, St. Louis, Mo.) in 96 well polystyrene microtiter plates. After 24 h, the plantonic cells were discarded and the biofilms washed with PBS. 0.10 ml of THB-10% horse serum was then added and the biofilms incubated for one h to induce maximum competence. Plasmid pPGS479 was then added and the biofilms incubated for an additional two hours prior to suspension and sonication of the biofilms followed by plating on Mitis salivarius-Erm agar plates.

### Biofilm formation

Biofilm formation was evaluated in 48-well polystyrene plates essentially as recently described [7]. For mixed biofilms, *S. gordonii *Challis or the BYW1 mutant were initially incubated in THB-0.1% glucose for 24 h, the resultant biofilms washed with PBS and incubated with *S. mutans *GS5 cells in THG-0.5% sucrose for an additional 24 h. The biofilms were washed, sonicated to disperse the cells, and serially diluted for plating on Mitis-salivarius agar plates to differentiate the *S. mutans *and *S. gordonii *cells.

## Results

### *S. gordonii *attenuates genetic transformation of *S. mutans*

Recent results in our laboratory have demonstrated that the presence of *S. gordonii *Challis, as well as other nonmutans oral streptococci, inhibited bacteriocin production by *S. mutans *GS5 and BM71 in broth cultures as well as in biofilms [7]. Such attenuation was mediated by the challisin protease secreted by strain Challis which degraded the competence stimulating peptide, CSP, a quorum sensing regulator in *S. mutans *which is required for bacteriocin production. In addition, the presence of strain Challis also markedly inhibited genetic transformation of *S. mutans *strains as exemplified for LT11 (Table 1). Such inhibition was also demonstrated for *S. mutans *strains GS5, NG8 and BM71 (data not shown). Despite the fact that CSP is also required for maximal transformation of *S. mutans*, mutants of strain Challis which do not express the challisin also inhibited transformation (data not shown). This suggested that in addition to attenuating the expression of the *S. mutans *CSP, *S. gordonii *also inhibited transformation of *S. mutans *via a CSP-independent mechanism. Furthermore, other oral streptococci such as *S. sanguis, S. mitis*, and *S. oralis *also were capable of inhibiting the transformation of *S. mutans *(data not shown).

### *S. gordonii *attenuates biofilm formation by *S. mutans*

Since *S. gordonii *appears to be one of the earliest colonizers of human teeth [9], it was of interest to determine if the presence of these organisms could influence subsequent biofilm formation by *S. mutans*. Preformed biofilms of *S. gordonii *Challis were established in microtiter plates, washed, and *S. mutans *GS5 was allowed to colonize layers of strain Challis in the presence of sucrose (Table 2). *S. mutans *GS5 did not colonize on *S. gordonii *as well as on the polystyrene plate surfaces (data not shown). Interestingly, strain GS5 colonized at higher rates on the challisin mutant, BYW1, than it did on the parental Challis strain. This suggests that attenuation of *S. mutans *CSP levels by the *S. gordonii *challisin protease inhibited sucrose-dependent biofilm formation by strain GS5. Thus, in addition to *S. mutans *GS5 bacteriocin production and transformation, *S. gordonii *also inhibits biofilm formation by the former strain.

## Discussion

The present results suggest that *S. gordonii*, and perhaps additional nonmutans streptococci, can attenuate some of the virulence properties of *S. mutans*, in part, by altering the quorum sensitive dependent properties of the later organisms. The recent demonstration that *S. gordonii *Challis can inactivate the CSP of *S. mutans *via a protease-dependent mechanism [7] as well as the documented role of this signaling molecule in biofilm formation, genetic transformation, aciduricity [10] and bacteriocin production (Yonezawa and Kuramitsu, in press) by *S. mutans *supports such a model. In addition, quorum-sensing independent effects of strain Challis on *S. mutans *GS5 were also demonstrated.

Sucrose-dependent biofilm formation by *S. mutans *strains appears to be an important virulence property of these organisms [2,11]. Furthermore, the present investigation demonstrated that the presence of *S. gordonii *appears to inhibit sucrose-dependent biofilm formation. The observation that the Challis mutant BYW1 did not inhibit this process as much as did the parental Challis strain suggests that CSP production by strain GS5 plays a significant role in sucrose-dependent biofilm formation. Such a role has recently been demonstrated in several strains of *S. mutans *when glucose is the primary carbon source [10,12]. However, the molecular basis for the role of CSP in *S. mutans *biofilm formation has not yet been determined and is currently under investigation in this laboratory.

Genetic transformation of *S. mutans *may not only serve as a means for expanding the genetic capabilities of this organism but could also have evolved as a means of scavenging essential purine and pyrimidine nutrients. Therefore, it may be possible that the attenuation of this property could alter the survivability of these organisms under conditions of nutrient limitation. Thus, the inhibition of *S. mutans *transformation by *S. gordonii *may provide the latter organism a competitive advantage in plaque where nucleotide precursors are limiting.

It is also possible that the presence of nonmutans streptococci such as *S. gordonii *could alter other quorum sensing-dependent properties of *S. mutans *including aciduricity, but this has not yet been examined. In addition, recent results in this laboratory have suggested that the sensitivity of strain GS5 to antimicrobial agents is also dependent upon CSP expression (Nakano and Kuramitsu, unpub. results). Therefore, the presence of *S. gordonii *may also increase the sensitivity of *S. mutans *in dental plaque to endogenous or exogenously applied antimicrobial agents.

## Conclusion

Taken together, the present results suggest that the presence of nonmutans streptococci in dental plaque could modulate the virulence properties of *S. mutans*. It will be of interest to subsequently determine if such interactions also occur *in vivo *and can modulate the cariogenicity of individual plaque samples. In addition, these results suggest potential novel strategies to attenuate the cariogenicity of *S. mutans *in plaque samples. These might include the development of antagonists of CSP activity or probiotic approaches involving commensal oral organisms which might attenuate *S. mutans *CSP activity.

## Competing interests

The author(s) declare that they have no competing interests.

## Authors' contributions

All authors read and approved the final manuscript.

## Figures and Tables

**Table 1:** Effects of *S. gordonii *Challis on *S. mutans *transformation. *S. mutans *LT11,*S. gordonii *Challis, or their mixtures were transformed with pPGS749 in either broth cultures or biofilms. Data are the mean +/- standard deviations of two independent experiments. ^a ^*S. mutans *LT11/*S. gordonii *Challis

	Transformants (10^7 ^cells)	
Cells	Broth	Biofilms
*S. mutans*	80.7 +/-44.5	347.4 +/-222.6
*S. gordonii*	1273.8 +/-402.3	2843.1 +/-1298.2
*S. mutans *+ *S. gordonii*	4.3 +/-5.4/1017.2 +/-261.1^a^	0/2716.2 +/-974.8

**Table 2:** *S. mutans *GS5 biofilm formation on preformed *S. gordonii *biofilms. Mixed biofilms were formed as described under Materials and Methods. Total cell numbers of *S. mutans S. gordonii *(x10^5^) were determined following dilution of the biofilm cells to three different levels. Data are the mean ± standard deviation of duplicate platings from one of two reproducible experiments.

Dilution	GS5/Challis	GS5/BYW1
x 1000	11.34 ± 2.46/26.54 ± 4.56	20.50 ± 3.56/23.25 ± 6.88
x 2000	1.04 ± 0.18/36.25 ± 5.82	9.18 ± 2.62/17.78 ± 7.44
x 4000	0.26 ± 0.04/41.5 ± 4.96	4.24 ± 1.96/32.75 ± 4.16
